# *Notes from the Field:* First Evidence of Locally Acquired Dengue Since 1944 — Guam, 2019

**DOI:** 10.15585/mmwr.mm6913a4

**Published:** 2020-04-03

**Authors:** Stephanie Kern-Allely, Ann Pobutsky, W. Thane Hancock

**Affiliations:** ^1^Council of State and Territorial Epidemiologists Fellowship Program; ^2^Guam Department of Public Health and Social Services; ^3^Division of State and Local Readiness, Center for Preparedness and Response, CDC.

On September 9, 2019, a resident of Guam with no travel history experienced a dengue-like illness that was reported to the Guam Department of Public Health and Social Services (DPHSS). On September 10, 2019, the Guam Public Health Laboratory (PHL) detected dengue virus 3 (DENV-3) in the patient’s serum specimen by reverse transcription–polymerase chain reaction (RT-PCR). This was the first detection of a locally acquired dengue case on Guam since 1944 (*1*). On September 11, Guam DPHSS initiated enhanced surveillance for suspected dengue cases and distributed a health alert to all health care providers with instructions for receiving dengue testing at the Guam PHL. On September 13, the Government of Guam declared a state of emergency to assist Guam DPHSS (*2*). Primary emergency response efforts included visits to homes within a 656-ft (200-m) radius of the primary residence of persons with confirmed locally acquired cases to provide educational materials, conduct case finding, implement mosquito source reduction, set traps for mosquito surveillance, and apply pesticides at homes of consenting residents. Public education efforts included billboards, pamphlets, and educational sessions held in schools and other community areas at risk. Updates on the clinical management of dengue using guidelines from CDC* and the World Health Organization (*3*) were delivered to all hospitals, medical societies, and most outpatient clinics.

A suspected case of dengue was defined as febrile illness in a Guam resident accompanied by at least two of the following signs or symptoms: myalgia, headache, arthralgia, eye or retro-orbital pain, rash, or hemorrhagic manifestations. A confirmed case was defined as any suspected case with laboratory confirmation of dengue virus infection by RT-PCR or anti-DENV immunoglobulin M (IgM) enzyme-linked immunosorbent assay (ELISA) (*4*).

During September 9–November 25, a total of 249 suspected cases were identified. Serum samples from 213 patients were submitted for RT-PCR testing at the Guam PHL. Diagnostic testing by IgM ELISA was conducted off-island by private clinical laboratories for 124 suspected cases, including 93 that had RT-PCR testing and 31 that did not. Among cases tested, 17 (7%) were confirmed, including 13 locally acquired and four travel-associated cases. Eleven of the locally acquired cases were RT-PCR–confirmed as DENV-3, and two were serologically confirmed. Onset dates of confirmed locally acquired cases occurred during September 3–November 11 (Figure). The median age of patients with locally acquired dengue was 12 years (range = <1–67 years) and 62% were male; two patients were hospitalized. Both suspected and confirmed cases were concentrated in the more densely populated northern and central regions of the island.

**FIGURE:**
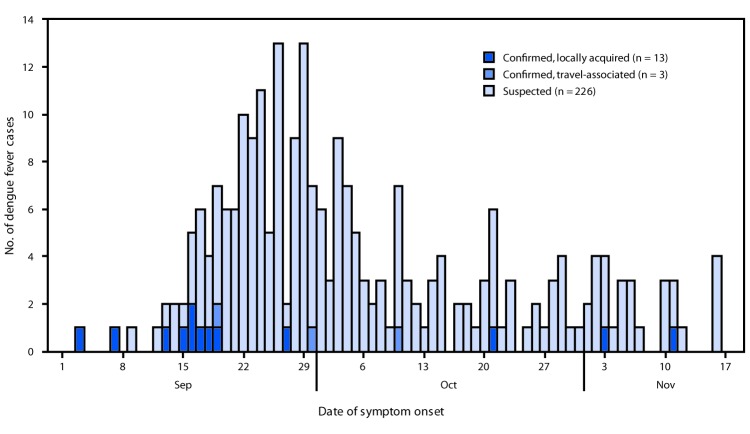
Number of confirmed*^,†^ and suspected^§^ cases of dengue fever, by date of symptom onset**^¶^** and source of infection (N = 242**) — Guam, September 3–November 16, 2019 * Confirmed cases that had symptom onset dates of September 18, September 27, and October 10, 2019, had positive results by immunoglobulin M (IgM) testing; all other confirmed cases had positive results by reverse transcription–polymerase chain reaction (RT-PCR). ^†^ Two confirmed cases had both positive RT-PCR and IgM results. ^§^ All suspected cases had negative results by RT-PCR or IgM testing ^¶^ Some illness onset dates have been estimated from date of specimen collection or date of laboratory results. ** One travel-associated case was identified during the surveillance period by IgM testing but had symptom onset before the surveillance period began; six suspected cases had not completed RT-PCR or IgM testing during the period and are not included.

Dengue outbreaks occur regularly in the Pacific Islands and outbreaks of DENV-3 are currently occurring in the Marshall Islands, Palau, the Philippines, and Yap State of the Federated States of Micronesia (*5*). Guam encounters imported dengue cases nearly annually because of frequent travel to and from Guam and areas with active DENV transmission. During 1988–2018, 42 cases of dengue were reported in Guam (*6*), and in 2019, before the outbreak in September, an additional three cases were reported (Guam Department of Public Health and Social Services, unpublished data, 2019); all 45 reported cases were travel-associated, most commonly from the Philippines (69%).

The last reported locally acquired dengue cases on Guam in 1944 involved *Aedes aegypti* as the vector (*1*). After the dengue outbreak and a subsequent outbreak of Japanese encephalitis in 1947, the U.S. military launched extensive vector control operations on the island, successfully eradicating *A. aegypti* (*1*). Current mosquito surveillance on Guam has identified *Aedes albopictus*, another DENV vector, but not *A. aegypti*. This outbreak provides evidence that autochthonous transmission of DENV is possible on Guam and likely transmitted by *A. albopictus*. It is important that future arboviral preparedness addresses gaps in detection and response exposed by the reemergence of dengue on Guam.

## Footnotes

* https://www.cdc.gov/dengue/resources/dengue-clinician-guide_508.pdf.
